# How to Apply Feedback to Improve Subjective Wellbeing of Government Servants Engaged in Environmental Protection in China?

**DOI:** 10.1155/2018/8529653

**Published:** 2018-02-14

**Authors:** Zhenxing Gong, Xinmeng Wang, Na Zhang, Miaomiao Li

**Affiliations:** ^1^School of Business, Liaocheng University, Liaocheng 252000, China; ^2^School of Economics and Management, Beijing Information Science and Technology University, Beijing 100192, China; ^3^Donglinks School of Economics and Management, University of Science and Technology Beijing, Beijing 100083, China

## Abstract

**Background:**

In order to improve subjective wellbeing of government servants engaged in environmental protection who work in high power distance in China, it is important to understand the impact mechanism of feedback. This study aims to analyze how feedback environment influences subjective wellbeing through basic psychological needs satisfaction and analyzing the moderating role of power distance.

**Method:**

The study was designed as a cross-sectional study of 492 government servants engaged in environment protection in Shandong, China. Government servants who agreed to participate answered self-report questionnaires concerning demographic conditions, supervisor feedback environment, basic psychological need satisfaction, and power distance as well as subjective wellbeing.

**Results:**

Employees in higher levels of supervisor feedback environment were more likely to experience subjective wellbeing. Full mediating effects were found for basic psychological needs satisfaction. Specifically, supervisor feedback environment firstly led to increased basic psychological needs satisfaction, which in turn resulted in increased subjective wellbeing. Additional analysis showed that the mediating effect of basic psychological needs satisfaction was stronger for employees who work in high power distance than in low power distance.

**Conclusion:**

The results from the study indicate that supervisor feedback environment plays a vital role in improving subjective wellbeing of government servants engaged in environmental protection through basic psychological needs satisfaction, especially in high power distance.

## 1. Introduction

Subjective wellbeing refers to how people experience the quality of their life [1] and is a public health concern associated with sociodemographic characteristics, health risks, stress, poor health, separation, unemployment, and lack of social contact consequences for the employee, as well as for the organization [2–4]. For the individual, subjective wellbeing has been found to lead to job burnout and turnover intention [5, 6]. Previous researchers have demonstrated the link between wellbeing and job outcomes such as commitment [7]. In recent years, the haze weather occurs frequently in China, and the whole society has paid close attention to the air quality problem. The pressure of environment protection makes environmental protection civil servants often have to work overtime, leading to work-family conflict increase. Inequality between rights and responsibilities results in the increase of helplessness, thus leading to a decline in subjective wellbeing. To facilitate environmental protection government servants' subjective wellbeing is therefore of importance.

The effects of environment variables can be felt in short period, but the demographic and personal characteristics are not likely to get intervention [8, 9]. Thus the environment factors have been a growing topic of organizational concern [10], and feedback is an important environment factor that impacts subjective wellbeing [11]. Considerable evidence indicates that positive feedback and negative feedback may result in subjective wellbeing [12, 13], and mixed feedback (including positive and negative feedback) is positively related to subjective wellbeing [14]. It is hard to know how to use feedback to improve subjective wellbeing; thus finding how the feedback influence subjective wellbeing becomes the problem that we should solve in this research.

What are the reasons for the inconsistency between subjective wellbeing and feedback and how to solve these problems?

First, feedback is taken as a dyadic method and compares the difference in one perspective. For example, according to valence, feedback can be divided into positive feedback and negative feedback. Some research found that positive feedback can improve subject wellbeing and negative feedback cannot, but some research found that negative feedback can improve subjective wellbeing if the feedback is accurate [12]. The feedback itself cannot be the former dependent variable, and the feedback's effects can be found with the interaction with feedback sources, feedback accuracy, and so on [15]. Previous studies have ignored the feedback recipients' construction, and the two-way communication effectiveness is not considered for the feedback of the recipient. In fact, the feedback behavior is interwoven with the feedback source, the feedback information, and the accuracy of the feedback, which is regulated by the individual to the feedback construction, and jointly influences the feedback effect [16]. Supervisor feedback environment has been pointed out as a crucial feature of subjective wellbeing management, which encompasses the contextual aspects of informal feedback process between the supervisor and subordinate [17]. Supportive supervisor feedback environment can lead to healthy performance systems; in this study we focus on the effects of supervisor feedback environment similar to previous studies [18].

Second, the existing research of how feedback impacts subjective wellbeing is not empirically supported [19]. Although prior research has demonstrated consistent relationships between feedback and subjective wellbeing [20, 21], it is not clear how feedback impacts subjective wellbeing. With respect to motivation mechanism, self-determination theory (SDT) put forward the fundamental theory which shows how external environments impact on internal motivation and in turn resulted in behavior [22]. Considering the formation of subjective wellbeing, SDT has shown the importance of basic psychological needs satisfaction [23], which includes autonomy, relatedness, and competence needs satisfaction and refers to essential experience for wellbeing [22]. Also, research has demonstrated that supporting environment can positively impact on basic psychological need satisfaction [23]. Thus, basic psychological needs satisfaction plays a mediation role between feedback environment and subjective wellbeing.

Third, prior research on feedback has focused mainly on constructive feedback or supportive feedback which is always beneficial for subjective wellbeing in US [8, 24] which has low power distance environment. China tends to be a high power distance country [25]; how feedback environment influences subjective wellbeing in high power distance country is not clear. Power distance refers to the extent to which people expect and accept that power is distributed unequally among persons and across different levels of the organizational hierarchy [26]. Because power distance can moderate the relationship between the feeling of empowerment and satisfaction [27], this finding would explore the moderation effect of high power distance on the influence of subjective wellbeing in question.

The aim of this study is to solve above problems by exploring how supportive supervisor feedback environment firstly led to basic psychological needs satisfaction, which in turn resulted in increased subjective wellbeing, and analyzing the moderating role of power distance.

## 2. Methods

### 2.1. Participants

A total of 492 questionnaires were distributed, and 426 usable questionnaires were returned (efficiency response rate of 87%). Of the employees, 55% (*n* = 235) were male and 45% (*n* = 191) were female. As for their age, 52% (*n* = 222) were aged 20–30 years, and 94% (*n* = 400) were under 40 years. With regard to their organizational tenure, 45% (*n* = 192) had worked for less than 5 years and 37% (*n* = 158) for 5–10 years (exclusive). 69% (*n* = 294) held a bachelor's degree or above.

### 2.2. Design and Data Collection

The study was designed as a cross-sectional study of 492 government servants engaged in environment protection in Shandong China. They come from different cities in Shandong Province. As Shandong Province is in the heavy area of China's smog, civil servants working in environmental protection in Shandong Province have become the object of our investigation. As consulting the agreement of leader, all the samples completed the questionnaires during their work hours. Participants were told that the aim of this study was to learn more about government servants. Participants need not write their name and this was voluntary without negative consequences.

Government servants who agreed to participate answered self-report questionnaires concerning demographic conditions, supervisor feedback environment, basic psychological need satisfaction, and power distance as well as subjective wellbeing.

*Supervisor Feedback Environment*. We measured the supervisor feedback environment using Steelman et al.'s scale [17]. This Likert scale assesses each feedback environment dimension and the seven facets within each dimension (32 items in total): source credibility, feedback quality, feedback delivery, accuracy of favorable feedback, accuracy of unfavorable feedback, source availability, and promoting feedback seeking. Cronbach's *α* for supervisor feedback environment was .93.

*Basic Psychological Need Satisfaction.* To assess basic psychological need satisfaction, we used a 9-item measure from Sheldon et al. [28], which taps into the satisfaction of autonomy and competence. An item from the basic psychological need satisfaction scale is as follows: “I feel a sense of choice and freedom in the things I undertake.” Items were rated on a 5-point Likert scale. Cronbach's alpha for basic psychological need satisfaction was .89.

*Power Distance.* The power distance scale was taken from the Robertson and Hoffman study [29]. The items were measured on a 7-point Likert scale ranging from (1) completely disagree to (7) completely agree. An example item is as follows: “managers should make most decisions without consulting subordinates.” For the power distance scale, Cronbach's alpha coefficient was .85.

*Subjective Wellbeing.* In line with past research [1], the Satisfaction with Life Scale [30] was used to evaluate participants' life satisfaction. The Satisfaction with Life Scale includes five questions and has been used in China context well [31]. This 5-item scale assesses participants' level of satisfaction with their life in general using a 7-point Likert-type response scale. Cronbach's alpha for subjective wellbeing was .91.

*Controls*. We controlled for sociodemographic differences including gender, education, organizational tenure, and age. We also controlled for coworker feedback environment using a measure from Steelman et al.'s scale [17], because the perception of coworker feedback environment may influence employee's ongoing feedback exchanges and satisfaction [17].

### 2.3. Statistical Analysis

Pearson correlation analysis was used to determine the relationships among the supervisor feedback environment, basic psychological need satisfaction, power distance, and subjective wellbeing. Regression analysis was used to determine the proportion of variance using SPSS 22.0. To test mediation, we adopted the procedure proposed by Preacher and Hayes [32]. Before the analyses, all continuous predictors were well-centered. To calculate the indirect effects, this study utilized the SPSS macro PROCESS [33]. To test the moderation, this study examined 3 conditions [32]: (a) significant effect of supervisor feedback environment on subjective wellbeing; (b) significant interaction between supervisor feedback environment and power distance in predicting subjective wellbeing; (c) different conditional indirect effect of supervisor feedback environment on subjective wellbeing, across low and high levels of power distance. To further validate findings of moderation relationships, we utilized an SPSS macro designed by Preacher and Hayes [32].

## 3. Results

Table 1 presents the means, standard deviations, and correlations among the study variables. An inspection of the correlations reveals that supervisor feedback environment was positively related to basic psychological needs satisfaction (*r* = .55, *p* < 0.01) and subjective wellbeing (*r* = .36, *p* < 0.01). The results also indicate that basic psychological needs satisfaction is positively correlated with the subjective wellbeing (*r* = .42, *p* < 0.01) and power distance (*r* = .18, *p* < 0.01).

To examine whether basic psychological needs satisfaction acted as a mediator of the relations between supervisor feedback environment and subjective wellbeing and whether power distance acted as a moderator of the relations between supervisor feedback environment and subjective wellbeing, we adopted the procedure proposed by Preacher and Hayes [32]. As shown in Table 2, after controlling for the effect of participant demographics, supervisor feedback environment significantly predicted basic psychological needs satisfaction (coeff = .69; 95% CI: .53–.84; *p* < 0.01) and subjective wellbeing (coeff = .57; 95% CI: .34–.80; *p* < 0.01). After the effect of the supervisor feedback environment toward subjective wellbeing was controlled, basic psychological needs satisfaction significantly predicted subjective wellbeing (coeff = .39; 95% CI: .16–.62; *p* < 0.01), power distance significantly predicted subjective wellbeing (coeff = −.35; 95% CI: .05–.52; *p* < 0.05), and the interaction between supervisor feedback environment and power distance also significantly predicted subjective wellbeing (coeff = .37; 95% CI: .06–.67; *p* < 0.05).

To calculate the indirect effects, we adopted the SPSS micro PROCESS [33]. Results in Table 3 show that the formal two-tailed significance test (assuming a normal distribution) demonstrated that the indirect effect was significant (Sobel *z* = 3.15, *p* < 0.01). Bootstrap results confirmed the Sobel test, with bootstrap 95% confidence interval of .11 to .47 around the indirect effect not containing zero. Taken together, supervisor feedback environment impacts subjective wellbeing via basic psychological needs satisfaction.

To further validate findings of moderation relationships, we used Preacher et al.'s (2007) statistical significance test, to compute a *z* statistic for the conditional direct effect. More specifically, we operationalized high and low levels of power distance as one standard deviation, such as above the variable's mean score one standard deviation and below the variable's mean score one standard deviation. Results in Table 4 show that the conditional direct effect of supervisor feedback environment was stronger and significant in the high power distance (conditional indirect effect = .75, SE = .15, *z* = 3.26, and *p* < 0.01, with 95% confidence interval of .20 to .83) and in moderate level of power distance (conditional indirect effect = .56, SE = .12, *z* = 2.01, and *p* < 0.05, with 95% confidence interval of .01 to .55) but was stronger and not significant in the low power distance condition (conditional indirect effect = 0.36, SE = .16, and *z* = 0.21, ns, with 95% confidence interval of −.32 to .40).

**Figure 1:**
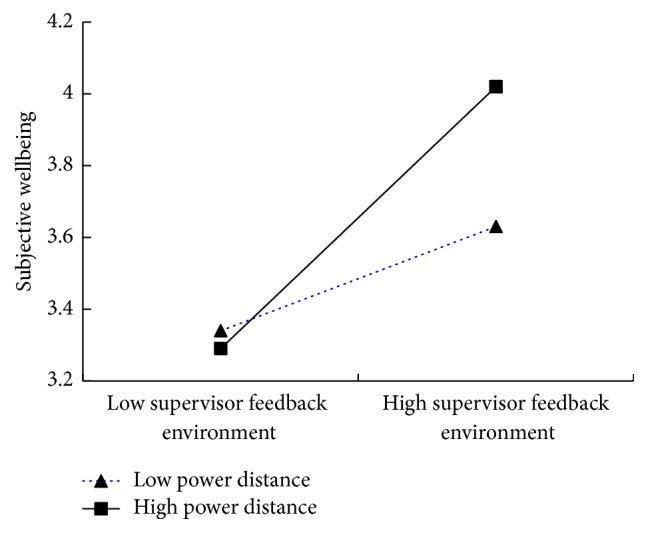
Simple slopes of supervisor feedback environment predicting subjective wellbeing at low (1 SD below M) and high (1 SD above M) levels of power distance.

The results in Figure 1 show that the conditional direct effect of supervisor feedback environment was stronger and more significant in the high power distance and was not significant in the low power distance. In sum, power distance acted as a moderator of the relations between supervisor feedback environment and subjective wellbeing.

## 4. Discussion

Our results indicate that employees in higher levels of supervisor feedback environment were more likely to experience subjective wellbeing. Full mediating effects were found for basic psychological needs satisfaction. Specifically, supervisor feedback environment firstly led to increased basic psychological needs satisfaction, which in turn resulted in increased subjective wellbeing. Additional analysis showed that the mediating effect of basic psychological needs satisfaction was stronger for employees who work in high power distance than in low power distance.

The positive relationship between perceived supervisor feedback environment and subjective wellbeing underscores the importance of providing feedback from a credit source, perceived as accuracy and availability, and encouraging feedback seeking. Basic psychological needs satisfaction mediates the relationship between supervisor feedback environment and subjective wellbeing; particularly the relationship between supervisor feedback environment and subjective wellbeing is stronger for employee in the higher power distance. This indicates that for the employee who works in China context, because of the high power distance in organization, supervisor feedback environment is more important than the employee who works in US context.

Supervisor feedback environment had a significant effect on subjective wellbeing after controlling of the effects of demographics. This finding is consistent with Alcantara et al., who found that social support and favorable developmental contexts can positively influence subjective wellbeing [34]. Prior research also found that support was a significant predictor for positive affect and satisfaction [35]. Supervisor feedback environment would be beneficial for a broader conceptualization of psychological empowerment as included in the current study based on self-determination theory in particular [22]. Research has demonstrated that supportive feedback environment would contribute to outcomes like performance and wellbeing [20, 36, 37]. Supportive supervisor feedback environment may motivate employees to work in a more positive way and increase the internal motivation of dealing with the conflict between job and family, so the employees can feel more satisfaction [21].

Basic psychological needs satisfaction had a significant direct effect on subjective wellbeing and full mediating effects were found for basic psychological needs satisfaction between supervisor feedback environment and subjective wellbeing. These effects are consistent with Çankaya, who demonstrated that there was a relationship between support autonomy and psychological needs satisfaction related to subjective wellbeing [38]. Like support autonomy, supervisor feedback environment plays a vital role in basic psychological needs satisfaction and subjective wellbeing.

This study found that the interaction term for supervisor feedback environment with power distance significantly would influence subjective wellbeing. A good relationship with supervisor is more important for job satisfaction in high power distance cultures. For an employee who works in high power distance, supervisor feedback environment can give subordinate effective and available information which can satisfy subordinate's basic need; specifically, supportive feedback environment lets the employees feel more favorable for supervisor [21]. Thus, the positive relationship between supervisor feedback environment and subjective wellbeing is stronger in high power distance work.

Based on this study, supervisor should strive to build favorable feedback environment and give feedback with more consideration of employees' basic psychological needs. It is important not only to achieve flow of top-down feedback but also to ensure feedback in parallel communication. Managers should create a high-support and high-care working atmosphere. Similar to basic human needs, self-determination theory posits that psychological needs must be met to promote optimal levels of wellbeing and, in work contexts, performance. Under the guidance of people-oriented principles, the management of environment protection government servants may consider reforming to allow environment protection government servants to have a greater degree of self-determination in their duties and provide more opportunities to the officers to participate in decision-making about environment protection.

There are two limitations. One limitation is investigation method. This research has adopted a variable-centered approach like prior research test feedback environment. A variable-centered approach assumes that employee perceives each dimension of feedback environment equally. In fact, different dimension plays different role in feedback environment. A person-centered approach can explore insights into feedback environment by using latent profile investigation and, specifically, can understand the typologies of supervisor feedback environment. Second, although we used a time-lag design to examine the role of feedback environment, the results of current research only concern the short-term outcomes. The developing of subjective wellbeing is a dynamic process like feedback. The employee who has higher level of subjective wellbeing will show more positive affect to supervisor; then supervisor is likely to give more positive feedback. Future study can use dynamic approach to study how subjective wellbeing influences feedback environment.

## 5. Conclusion

The results from the study indicate that supervisor feedback environment plays a vital role in improving subjective wellbeing of government servants engaged in environmental protection through basic psychological needs satisfaction, especially in high power distance. Leaders who work in environmental protection agency should pay more attention to strive to build a supportive feedback environment for improving subjective wellbeing of government servants engaged in environmental protection.

## Figures and Tables

**Table 1:** Means, standard deviations, and correlations of all measures.

	Mean	SD	1	2	3	4
(1) Supervisor feedback environment	3.42	.50	-			
(2) Power distance	3.31	.65	−.04	-		
(3) Basic psychological needs satisfaction	3.43	.62	.55^*∗∗*^	.18^*∗*^	-	
(4) Subjective wellbeing	3.63	.79	.36^*∗∗*^	−.10	.42^*∗∗*^	-
(5) Gender	-	-	−.01	−.04	−.12	−.12
(6) Age	2.17	1.07	−.07	.01	.11	.02
(7) Job tenure	3.22	1.55	.01	−.01	.15^*∗*^	.03
(8) Education	-	-	.03	.02	−.14	−.13

*Note*. *n* = 426; ^*∗*^*p* < 0.05 and ^*∗∗*^*p* < 0.01.

**Table 2:** Hierarchical regression results about mediation and moderation effect.

	Basic psychological needs satisfaction as dependent variable			Subjective wellbeing as dependent variable			Subjective wellbeing as dependent variable		
	Coeff	95% CI	*p*	coeff	95% CI	*p*	Coeff	95% CI	*p*
Gender	−.12	−.28–.05	0.16	−.18	−.41–.06	0.14	−.15	−.38–.08	0.19
Age	.03	−.14–.21	0.70	.05	−.20–.30	0.71	.01	−.23–.25	0.94
Job tenure	.04	−.08–.15	0.55	−.02	−.19–.16	0.83	−.03	−.19–.14	0.76
Education	−.07	−1.44–.02	0.06	−.83	−.89–.22	0.12	−.05	−.58–.47	0.28
Supervisor feedback environment	.69^*∗∗*^	.53–.84	0.01	.57^*∗∗*^	.34–.80	0.01	.09	−2.01–.35	0.11
Basic psychological needs satisfaction							.39^*∗∗*^	.16–.62	0.01
Power distance							−.35^*∗*^	.05–.52	0.02
Supervisor feedback environment × power distance							.37^*∗*^	.06–.67	0.03
*R* ^2^	.35			.16			.24		
*F*	17.33^*∗∗*^			5.96^*∗∗*^			6.38^*∗∗*^		

*Note. n* = 426; ^*∗*^*p* < 0.05 and ^*∗∗*^*p* < 0.01. Coeff = standardized coefficients; CI = confidence interval.

**Table 3:** Results of Sobel test and bootstrapping for the indirect effect of supervisor feedback environment on subjective wellbeing via basic psychological needs satisfaction.

Test	Value	SE	*z*	*p*	LL 95% CI	UL 95% CI
Sobel test results for indirect effect	.27	.09	3.15	0.01	.12	.46
Bootstrap results for indirect effect	.27	.08	3.12	0.01	.11	.47

*Note*. *n* = 426. LL = lower limit; CI = confidence interval; UL = upper limit.

**Table 4:** Results for conditional direct effect of supervisor feedback environment on subjective wellbeing across levels of power distance.

Moderator level	Mean	Conditional indirect effect	SE	*z*	*p*	LL 95% CI	UL 95% CI
Low (M – 1 SD)	−.65	.36	.16	.21	0.83	−.32	.40
Moderate level	.00	.56	.12	2.01	0.05	.01	.55
High (M + 1 SD)	.65	.75	.15	3.26	0.01	.20	.83

*Note*. *n* = 426. LL = lower limit; CI = confidence interval; UL = upper limit.

## References

[1] Diener E., Suh E. M., Lucas R. E., Smith H. L. (1999). Subjective well-being: Three decades of progress. *Psychological Bulletin*.

[2] Yawson A. E., Baddoo A., Hagan-seneadza N. A. (2013). Tobacco use in older adults in Ghana: sociodemographic characteristics, health risks and subjective wellbeing. *BMC Public Health*.

[3] Harryson L., Aléx L., Hammarström A. (2016). "I have surly passed a limit, it is simply too much": Women's and men's experiences of stress and wellbeing when living within a process of housework resignation. *BMC Public Health*.

[4] Dolan P., Peasgood T., White M. (2008). Do we really know what makes us happy? A review of the economic literature on the factors associated with subjective well-being. *Journal of Economic Psychology*.

[5] Brunetto Y., Farr-Wharton R., Shacklock K. (2012). Communication, training, well-being, and commitment across nurse generations. *Nursing Outlook*.

[6] Rho M. J. U., Kim S. R. A., Kim H.-S. (2014). Exploring the relationship among user satisfaction, compliance, and clinical outcomes of telemedicine services for glucose control. *Telemedicine journal and e-health : the official journal of the American Telemedicine Association*.

[7] Wright T. A., Cropanzano R. (2000). Psychological well-being and job satisfaction as predictors of job performance.. *Journal of Occupational Health Psychology*.

[8] Gabriel A. S., Frantz N. B., Levy P. E., Hilliard A. W. (2014). The supervisor feedback environment is empowering, but not all the time: Feedback orientation as a critical moderator. *Journal of Occupational and Organizational Psychology*.

[9] Zhang J., Gong Z., Zhang S., Zhao Y. (2017). Impact of the supervisor feedback environment on creative performance: A moderated mediation model. *Frontiers in Psychology*.

[10] Suárez-Varela M., Guardiola J., González-Gómez F. (2016). Do Pro-environmental Behaviors and Awareness Contribute to Improve Subjective Well-being?. *Applied Research in Quality of Life*.

[11] Kluetsch R. C., Ros T., Théberge J. (2014). Plastic modulation of PTSD resting-state networks and subjective wellbeing by EEG neurofeedback. *Acta Psychiatrica Scandinavica*.

[12] Andiola L. M. (2014). Performance feedback in the audit environment: A review and synthesis of research on the behavioral effects. *Journal of Accounting Literature*.

[13] van der Rijt J., van de Wiel M. W. J., Van den Bossche P., Segers M. S. R., Gijselaers W. H. (2012). Contextual antecedents of informal feedback in the workplace. *Human Resource Development Quarterly*.

[14] Pawlowski C. A. Therapeutic effects of MMPI-2 feedback favorability on client subjective well -being: a process and outcome study.

[15] Dahling J., O'Malley A. L., Chau S. L. (2015). Effects of feedback motives on inquiry and performance. *Journal of Managerial Psychology*.

[16] Norris-Watts C., Levy P. E. (2004). The mediating role of affective commitment in the relation of the feedback environment to work outcomes. *Journal of Vocational Behavior*.

[17] Steelman L. A., Levy P. E., Snell A. F. (2004). The feedback environment scale: Construct definition, measurement, and validation. *Educational and Psychological Measurement*.

[18] Peng J.-C., Chiu S.-F. (2010). An integrative model linking feedback environment and organizational citizenship behavior. *The Journal of Social Psychology*.

[19] London M. (2003). Job feedback: Giving, seeking, and using feedback for performance improvement: Second edition. *Job Feedback: Giving, Seeking, and Using Feedback for Performance Improvement: Second Edition*.

[20] Rosen C. C., Levy P. E., Hall R. J. (2006). Placing perceptions of politics in the context of the feedback environment, employee attitudes, and job performance. *Journal of Applied Psychology*.

[21] Sparr J. L., Sonnentag S. (2008). Fairness perceptions of supervisor feedback, LMX, and employee well-being at work. *European Journal of Work and Organizational Psychology*.

[22] Deci E. L., Ryan R. M. (2000). The ‘what’ and ‘why’ of goal pursuits: human needs and the self-determination of behavior. *Psychological Inquiry*.

[23] Tian L., Chen H., Huebner E. S. (2014). The longitudinal relationships between basic psychological needs satisfaction at school and school-related subjective well-being in adolescents. *Social Indicators Research*.

[24] Madjar N., Oldham G. R., Pratt M. G. (2002). There's no place like home? The contributions of work and nonwork creativity support to employees' creative performance. *Academy of Management Journal (AMJ)*.

[25] Pan Q., Wei H. Influences of trust in subordinate, perceived risk and power distance on leader empowering behavior: Empirical study from China.

[26] Fock H., Hui M. K., Au K., Bond M. H. (2013). Moderation Effects of Power Distance on the Relationship Between Types of Empowerment and Employee Satisfaction. *Journal of Cross-Cultural Psychology*.

[27] Hui M. K., Au K., Fock H. (2004). Empowerment effects across cultures. *Journal of International Business Studies*.

[28] Sheldon K. M., Elliot A. J., Kim Y., Kasser T. (2001). What is satisfying about satisfying events? Testing 10 candidate psychological needs. *Journal of Personality and Social Psychology*.

[29] Robertson C. J., Hoffman J. J. (2000). How different are we? an investigation of confucian values in the united states. *Journal of Managerial Issues*.

[30] Diener E., Biswas-Diener R. (2002). Will money increase subjective well-being? A literature review and guide to needed research. *Social Indicators Research*.

[31] Liu Y., Zhang F., Wu F., Liu Y., Li Z. (2017). The subjective wellbeing of migrants in Guangzhou, China: The impacts of the social and physical environment. *Cities*.

[32] Preacher K. J., Hayes A. F. (2008). Asymptotic and resampling strategies for assessing and comparing indirect effects in multiple mediator models. *Behavior Research Methods*.

[33] Hayes A. F. (2013). Introduction to mediation, moderation, and conditional process analysis: A regression-based approach. *Journal of Educational Measurement*.

[34] Alcantara S. C., González-Carrasco M., Montserrat C., Viñas F., Casas F., Abreu D. P. (2016). Peer violence in the School Environment and Its Relationship with Subjective Well-Being and Perceived Social Support Among Children and Adolescents in Northeastern Brazil. *Journal of Happiness Studies*.

[35] Bojanowska A., Zalewska A. M. (2011). Subjective well-being among teenagers of different ages: the role of emotional reactivity and social support from various sources. *Studia Psychologiczne*.

[36] Jennifer L. S., Sabine S. (2008). Fairness perceptions of supervisor feedback, LMX, and employee well-being at work. *European Journal of Work & Organizational Psychology*.

[37] Whitaker B. G., Dahling J. J., Levy P. (2007). The development of a feedback environment and role clarity model of job performance. *Journal of Management*.

[38] Çankaya Z. C. (2009). Autonomy support, basic psychological need satisfaction and subjective well-being: self determination theory. *Turkish Psychological Counseling & Guidance Journal*.

